# Inconclusive mutually reinforcing effects between associative learning and fluid intelligence: simulated reanalyses and a comment on Ren et al. (2026)

**DOI:** 10.3389/fpsyg.2026.1821604

**Published:** 2026-08-07

**Authors:** Kimmo Sorjonen, Bo Melin, Marika Melin

**Affiliations:** Department of Clinical Neuroscience, Karolinska Institutet, Stockholm, Sweden

**Keywords:** associative learning, fluid intelligence, mutualism theory of intelligence, random-intercept cross-lagged panel model (RI-CLPM), spurious effects, statistical rigor

## Abstract

Based on findings from analyses with the random-intercept cross-lagged panel model (RI-CLPM), Ren et al. concluded reinforcing longitudinal effects between associative learning and fluid intelligence. However, the RI-CLPM is susceptible to spurious findings. Here, we analyzed data simulated to resemble the data used by Ren et al. with alternative models and found discrepant increasing, decreasing, and null prospective effects between associative learning and fluid intelligence. Hence, the conclusions by Ren et al. appear not to be supported by their own data. It is important for researchers to bear in mind that correlations, including effects in the RI-CLPM, in observational (i.e., non-experimental) data may be spurious and do, consequently, not prove genuine (i.e., non-spurious) influence. We recommend researchers to fit alternative models to data and to base conclusions on an aggregation of findings.

## The study by Ren et al.

Ren et al. (2026) analyzed data on associative learning and fluid intelligence, collected from elementary school children in China (*N* = 160) on three occasions, with random-intercept cross-lagged panel models (RI-CLPM, Hamaker et al., 2015; Mulder and Hamaker, 2021). Based on positive within-individual cross-lagged effects, Ren et al. (2026) concluded that associative learning (the ability to form and reorganize connections between information, supporting memory, concept formation, adaptive thinking, and knowledge integration) and fluid intelligence (the capacity for abstract reasoning and novel problem-solving, widely regarded as a core indicator of general cognitive ability) mutually reinforce each other’s longitudinal development. This conclusion was in line with the mutualism theory of intelligence, which posits that positive associations between different cognitive tests (the positive manifold) may be due to reciprocal causal reinforcing effects between different abilities rather than due to a common source, i.e., *g* (general intelligence) (Kievit et al., 2017; van der Maas et al., 2006). According to Ren et al. (2026), their study “expands the literature by employing a longitudinal design that enables examination of intraindividual changes over time, which cannot be assessed using cross-sectional data” (p. 7).

## Potential spuriousness and suggested counteractions

As described above, based on findings from analyses with the RI-CLPM, Ren et al. (2026) suggested effects in line with the mutualism theory of intelligence. However, we have previously challenged the study by Kievit et al. (2017), claiming support for the mutualism theory, and shown that their findings of prospective effects between vocabulary and matrix reasoning may have been spurious rather than due to mutual reinforcement (Sorjonen et al., 2023). Moreover, unless included as covariates in the model, RI-CLPM does not adjust for time-varying confounders (Murayama and Gfrörer, 2025; Rohrer and Murayama, 2023). This means that RI-CLPM, just like the traditional cross-lagged panel model, is susceptible to spurious findings. Consequently, the findings by Ren et al. (2026) may have been spurious and their conclusions premature.

Researchers, at least in psychology, often fit only one model to their data. In multiverse analyses, on the other hand, researchers fit several alternative models to data and base conclusions on an aggregation of findings (Steegen et al., 2016, see also Olsson-Collentine et al., 2025). We have suggested, in line with multiverse methodology, that findings from the RI-CLPM may be evaluated by fitting alternative models to data, e.g., time-reversed RI-CLPMs, latent change score models (LCSM, Ghisletta and McArdle, 2012; Kievit et al., 2018; McArdle, 2009), and multilevel models of person-mean centered scores. Using this methodology we have shown, for example, that prospective within-individual effects between executive functions and symptoms of psychiatric disorders (Sorjonen and Melin, 2025a), curiosity and creativity (Sorjonen and Melin, 2025b), and academic self-concept (i.e., self-perceived competence) and achievement (Sorjonen et al., 2025a,b) may have been spurious. These findings challenged more or less explicit suggestions of causal effects between these constructs in previous work. Here, we used the same methodology to scrutinize the findings and conclusions by Ren et al. (2026).

It may be noted that there is no specific connection between multiverse analyses and the RI-CLPM. Instead, multiverse methodology is a general term used for analyses where plausible models, e.g., regression or structural equation models, are fitted to the same data and conclusions are based on an aggregation of findings instead of, which is common in psychological research, only one of the models. This results in more robust findings that are less affected by researcher choices, which often may be quite arbitrary. If the effect of interest aligns across analyzed models, conclusions may be drawn with increased confidence. If, on the other hand, the effect differs between models, this suggests that the effect in any one of the models may be spurious (a statistical artifact) and that confident conclusions should be postponed.

The present simulations and analyses were conducted with R 4.4.3 statistical software (R Core Team, 2026) using the MASS (Venables and Ripley, 2002), psych (Revelle, 2020), and lavaan (Rosseel, 2012) packages. The analytics script, which also generates the simulated data, is available at the Open Science Framework at https://osf.io/e9t65/.

## Simulated reanalyses

**Figure 1:**
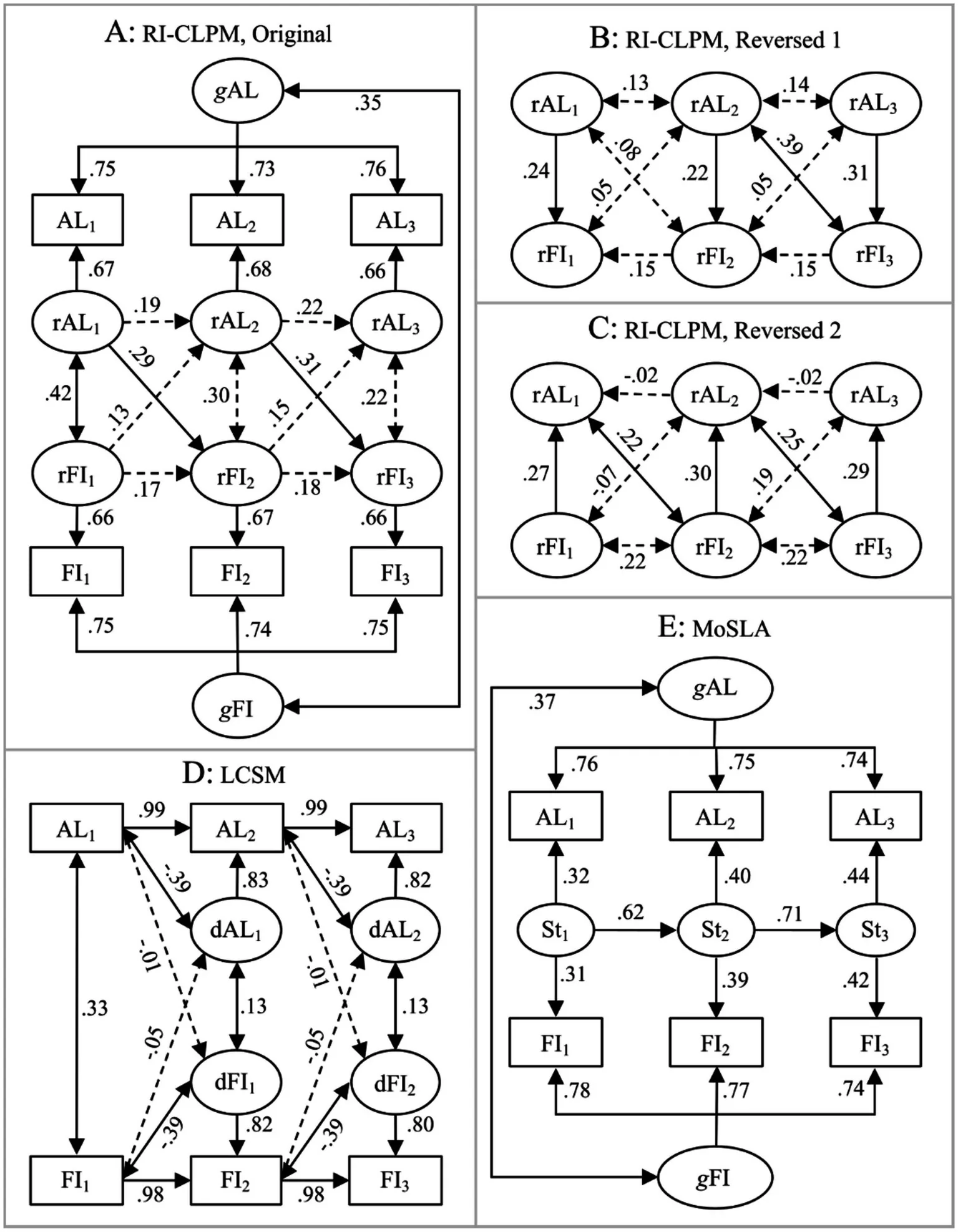
**(A)** A random-intercept cross-lagged panel model (RI-CLPM) with associative learning (AL) and fluid intelligence (FI), measured at three occasions, regressed on stable trait-like levels of the constructs (*gAL* and *gFI*, respectively). Auto-regressive and cross-lagged effects were estimated between within-individual residual levels of the constructs not accounted for by the stable trait-like levels (*rAL_1_*, *rFI_1_*, etc.); **(B)** A reversed RI-CLPM, where within-individual fluid intelligence was regressed on contemporaneous associative learning and subsequent fluid intelligence; **(C)** A reversed RI-CLPM, where within-individual associative learning was regressed on contemporaneous fluid intelligence and subsequent associative learning; **(D)** A latent change score model (LCSM), where scores on associative learning and fluid intelligence were regressed on previous scores on the constructs as well as latent change scores (e.g., *dAL_1_*). The latent change scores were, in turn, regressed on the previous score on the other construct; **(E)** A model of spurious longitudinal associations (MoSLA), where longitudinal scores on associative learning and fluid intelligence were regressed on stable trait-like latent variables (i.e., random intercepts) as well as auto-correlated state factors (e.g., *St1*). The model did not include any direct effects between scores on associative learning and fluid intelligence. Models **B** and **C** included, just like model **A**, random intercepts with effects on observed scores. For simplicity, we only present the within-individual part of the models here. Solid parameters were statistically significant (*p* < 0.05) while dashed parameters were not. All parameter-values were standardized.

We simulated data with the same sample size and correlations between variables as in the empirical data analyzed by Ren et al. (2026). We used simulated data as the empirical data were not available to us. The corresponding authors of the study by Ren et al. (2026) did not respond to our request for the data. However, it is important to note that regression effects, including effects and associations in structural equation models, are functions of correlations. Consequently, these effects and associations will be the same in data with the same correlations between variables, irrespective if the data is empirical or simulated (the analytic algorithms do not know if the data is empirical or simulated). Following Sorjonen and Melin (2025a, 2025b), we fitted the five models in Figure 1 to the simulated data:

(A) A RI-CLPM suggested, in line with findings by Ren et al. (2026), a positive within-individual cross-lagged effect of associative learning on subsequent fluid intelligence when adjusting for initial fluid intelligence (e.g., *β* = 0.293 [0.082; 0.504], *p* = 0.007). However, the cross-lagged effect of fluid intelligence on subsequent associative learning was not statistically significant (e.g., *β* = 0.131 [−0.093; 0.356], *p* = 0.251). The model had excellent fit (χ^2^ = 4.11, *DF* = 5, *p* = 0.533, CFI = 1.00, TLI = 1.01, RMSEA = 0.00 [90% CI: 0.00; 0.10]).
(B) A reversed RI-CLPM suggested a positive within-individual effect of associative learning on contemporaneous fluid intelligence when adjusting for subsequent fluid intelligence (e.g., *β* = 0.235 [0.027; 0.444], *p* = 0.027). This would mean that among individuals with the same within-individual score on subsequent fluid intelligence (e.g., 0), those with a higher score on initial associative learning (e.g., 1) were predicted to have had a higher score on initial fluid intelligence (e.g., 1 × 0.235 = 0.235) and, consequently, to have experienced a larger decrease in fluid intelligence between the measurements (0–0.235 = −0.235) compared with those with the same subsequent fluid intelligence but lower initial associative learning (e.g., −1 × 0.235 = −0.235 and 0 − (−0.235) = 0.235). This positive effect suggested that low initial associative learning may have counteracted low initial fluid intelligence and allowed individuals to reach the same subsequent fluid intelligence as individuals with higher initial fluid intelligence and higher initial associative learning. The model had excellent fit (χ^2^ = 1.42, *DF* = 5, *p* = 0.922, CFI = 1.00, TLI = 1.02, RMSEA = 0.00 [90% CI: 0.00; 0.04]).
(C) A second reversed RI-CLPM suggested a positive within-individual effect of fluid intelligence on contemporaneous associative learning when adjusting for subsequent associative learning (e.g., *β* = 0.273 [0.070; 0.476], *p* = 0.009). This would indicate, similarly as above, that low initial fluid intelligence may have counteracted low initial associative learning and allowed individuals to reach the same subsequent associative learning as individuals with higher initial associative learning and higher initial fluid intelligence. The model had excellent fit (χ^2^ = 2.51, *DF* = 5, *p* = 0.775, CFI = 1.00, TLI = 1.02, RMSEA = 0.00 [90% CI: 0.00; 0.07]).
(D) A latent change score model (LCSM) suggested no effects of initial associative learning on subsequent change in fluid intelligence (e.g., β = −0.011 [−0.096; 0.074], *p* = 0.799) or vice versa (e.g., β = −0.052 [−0.136; 0.033], *p* = 0.229). The model had excellent fit (χ^2^ = 11.3, *DF* = 13, *p* = 0.587, CFI = 1.00, TLI = 1.00, RMSEA = 0.00 [90% CI: 0.00; 0.07]).
(E) A model of spurious longitudinal associations (MoSLA), where longitudinal scores on associative learning and fluid intelligence were regressed on stable trait-like latent variables (i.e., random intercepts) as well as auto-correlated state factors, had excellent fit (χ^2^ = 12.2, *DF* = 15, *p* = 0.662, CFI = 1.00, TLI = 1.01, RMSEA = 0.00 [90% CI: 0.00; 0.06]). This suggested that data analyzed by Ren et al. (2026), and simulated and reanalyzed by us, may have been generated without any direct effects between scores on associative learning and fluid intelligence, i.e., that such effects may have been spurious.

We chose to fit models A–D to data as they may be used to estimate the focal effects of the present commentary, i.e., the effect of associative learning on subsequent change in fluid intelligence and vice versa. The original RI-CLPM (model A) and the LCSM (model D) are two well-established models for estimating prospective associations between constructs. The reversed RI-CLPM (models B and C), on the other hand, is consistent with a recommendation by Campbell and Kenny (1999) to use time-reversed analyses as a tool to identify statistical artifacts. With genuine prospective effects, time-reversal should reverse the sign of the effect. The stable trait, autoregressive trait, and state (STARTS) model (sometimes referred to as the trait–state-error (TSE) model) (Kenny and Zautra, 1995) may also be used to estimate prospective effects, but not in the present case as it requires data from at least four waves of measurement while the present data only included three waves. Moreover, we fitted the MoSLA (model E) to data in order to evaluate if the data may have been generated without direct effects between longitudinal scores on associative learning and fluid intelligence. The MoSLA assumes, just like the RI-CLPM, that longitudinal scores on constructs are affected by stable trait-like levels of the constructs. However, the MoSLA also assumes that contemporaneous scores on the two constructs are affected by common occasion-specific (although auto-correlated) state-factors. Both of these assumptions of the MoSLA are, in our opinion, plausible for many combinations of related constructs, e.g., associative learning and fluid intelligence.

## Summary and concluding remarks

Of five analyzed models, only the original RI-CLPM suggested positive, i.e., increasing, prospective within-individual effects between associative learning and fluid intelligence. Two reversed RI-CLPMs suggested paradoxical decreasing effects and a LCSM suggested no effects of associative learning on subsequent change in fluid intelligence and vice versa. The MoSLA suggested that data may have been generated without any direct effects between scores on associative learning and fluid intelligence. An aggregation of these discrepant findings indicates, in our opinion, that there may have been no genuine (i.e., non-spurious) reinforcing longitudinal effects between associative learning and fluid intelligence in the data analyzed by Ren et al. (2026). Hence, the conclusions by Ren et al. (2026) in this regard appear premature and can be challenged. In extension, because the mutualism theory of intelligence predicts genuine reinforcing longitudinal effects between different cognitive abilities, the present results suggest that the findings by Ren et al. (2026) do not offer support to the mutualism theory of intelligence.

It should be noted that the present commentary does not challenge the presence of positive prospective within-individual associations between associative learning and fluid intelligence in the data used by Ren et al. (2026) *per se*. These positive associations were verified by our analyses with the original RI-CLPM (Figure 1A). However, as shown, these positive associations were limited to a situation with statistical adjustment for an initial within-individual score on the outcome. Without this adjustment, findings suggested negative (Figures 1B,C) or no (Figure 1D) prospective associations. Instead of concluding that the data simultaneously supports claiming positive, negative, and null prospective associations, we prefer to recommend caution and to postpone claiming both positive and negative prospective associations.

It is important for researchers to bear in mind that correlations, including effects in the RI-CLPM, in observational (i.e., non-experimental) data may be spurious as well as due to genuine effects. We recommend researchers to, as we did here, fit alternative models to data. If effects in different models converge, conclusions can be drawn with increased confidence. If, on the other hand and as in the present case, effects diverge, caution is advised and confident conclusions in either direction should probably be suspended. This recommendation is consistent with multiverse methodology. The core point of this methodology is that researchers should not make more or less arbitrary choices between alternative models. Instead, all conceivable models providing an estimate of the effect or association of interest should be fitted to the data, unless convincing arguments why findings from a specific model lack informative value can be made. Then, conclusions should be based on an aggregation of the findings. For scrutiny of claimed prospective within-individual associations based on analyses with the RI-CLPM, the analytic script for the present commentary, available at https://osf.io/e9t65/, may be modified and used with other datasets.

## Data Availability

Publicly available datasets were analyzed in this study. This data can be found here: the analytics script, which also generates the simulated data, is available at the Open Science Framework at https://osf.io/e9t65/.
